# Death

**Published:** 1887-04-16

**Authors:** 


### Words of Consolation.

[Communications for this column should be addressed, "Chaplain," care of the Editor of The Hospital, who will gratefully welcome any suggestions or inquiries from matrons, nurses, and the staff generally.]

XXIX.—Death.

As we endeavour to bring our own views of death into harmony with the Gospel revelation, it is very necessary to guard against erroneous ideas which may arise from pressing the word "sleep" too far. One thing it is certainly not meant to teach us. It is not meant to teach us that our present state of life ends in a long period of unconsciousness, or that when the spirit passes from the body to Paradise it remains in darkness or oblivion. This much has sometimes been inferred from what is said in Holy Scripture of sleeping in death. But such an inference is to make Scripture contradict itself. We are taught in numerous passages (see Luke xvi, 19-31; 1 Pet. iii, 19 ; iv, 6 ; Rev. vi, 9-10) that the spirits of the departed are fully conscious, active, and in some mysterious possession of most of the senses they exercised in the body. The righteous are at rest, in so far as they are free from labour or trouble or pain ; and yet they are evidently not resting in such a way as to be unconscious of their surroundings. Be it remembered that ordinary sleep does not by any means imply unconsciousness. The mind is as active sometimes during sleep, although in a different way, as it is when we are awake. The intermediate state is (we can little doubt it) a state of realisation far exceeding any comparison which can be made with dreaming. It is as well, nevertheless, to bear in mind that here the soul is probably never quite a blank, even in the soundest sleep. No ; what "sleep" implies above all things is that it must be followed by an awakening. There is an *end* to sleep ; but there is no end to death, so far as we know. To say that a person is asleep, generally conveys your belief in his awakening in a very short time, rested, renewed, capable of doing all or more than he was before, because the body has put off its weariness, and become once more a ready exponent of the soul's desires. All this in the highest degree is the precious heritage of a sincere faith, as it looks through the sleep of the just to the dawn of a joyful resurrection. Far otherwise with the thought of "death." Death implies a separation far more terrible, a separation which will only be intensified in the awakening to judgment, for then death will be realised in its very essence as the condemnation of those who deliberately choose in this life to separate themselves from God. "Depart from me, ye cursed, into everlasting fire, prepared for the devil and his angels" (Matt. xxv, 41). Let us not shrink from analysing our views of death ; and if we find that far down in the silent depth of the soul there yet lurks some fear of the real death, it may be well. God will scarcely allow *that* to remain without a cause ; and the only cause possible is sin, which separates the soul from Him. Is it not a mercy that He should allow such fear so long as sin remaineth ?

(*To be continued.*)

